# Delays in care seeking, diagnosis and treatment of patients with pulmonary tuberculosis in Hubei, China

**DOI:** 10.1093/inthealth/ihz036

**Published:** 2019-06-22

**Authors:** Qin Yang, Yeqing Tong, Xiaoxv Yin, Lei Qiu, Na Sun, Yuxin Zhao, Dandan Li, Xiaotong Li, Yanhong Gong

**Affiliations:** School of Public Health, Tongji Medical College, Huazhong University of Science and Technology, Wuhan, P.R. China; Center for Disease Control and Prevention of Hubei Province, Wuhan, P.R. China; School of Public Health, Tongji Medical College, Huazhong University of Science and Technology, Wuhan, P.R. China; School of Public Health, Tongji Medical College, Huazhong University of Science and Technology, Wuhan, P.R. China; School of Public Health, Tongji Medical College, Huazhong University of Science and Technology, Wuhan, P.R. China; School of Public Health, Tongji Medical College, Huazhong University of Science and Technology, Wuhan, P.R. China; School of Public Health, Tongji Medical College, Huazhong University of Science and Technology, Wuhan, P.R. China; School of Public Health, Tongji Medical College, Huazhong University of Science and Technology, Wuhan, P.R. China; School of Public Health, Tongji Medical College, Huazhong University of Science and Technology, Wuhan, P.R. China

**Keywords:** care seeking, delay, diagnosis, treatment, tuberculosis

## Abstract

**Background:**

Early diagnosis and treatment are essential for effective tuberculosis (TB) control. However, delays in the diagnosis and treatment of TB in central China have not been sufficiently investigated. This cross-sectional study was conducted between October 2013 and March 2014 in Hubei, China to identify risk factors of delays in care seeking, diagnosis and treatment among patients with TB.

**Methods:**

A total of 1342 patients with TB seen in the designated institutions were included. Multivariate logistic regression was used to analyse factors associated with delays in TB diagnosis and treatment.

**Results:**

Overall, 21.54%, 23.62% and 42.25% of patients with TB experienced delays in care seeking, diagnosis and treatment, respectively. Multivariate logistic regression showed that medical insurance and monthly household income were significantly associated with delays in care seeking. The time to reach a township hospital or the facility of a patient’s first consultation was significantly associated with delays in diagnosis. Sex, education, time to reach a township hospital and the facility where the diagnosis was made were significantly associated with delays in treatment.

**Conclusions:**

Delays in TB diagnosis and treatment in Hubei remain a serious issue. Improvements in the capability and accessibility of health care services are imperative to reduce delays and expedite TB diagnosis and treatment.

## Introduction

Tuberculosis (TB) is a global public health problem. The latest global TB report shows that 10.4 million people fell ill with TB and 1.7 million died from the disease in 2016, imposing a heavy economic burden on families and society. China is one of 30 countries with a high burden of TB. In 2016, the prevalence of TB in China was ranked third highest worldwide.^1^

Early diagnosis and active treatment are key to effective prevention and control of TB.^2–4^ Between 2000 and 2016, an estimated 53 million patients with TB were saved worldwide through timely diagnosis and treatment.^1^ However, because the clinical symptoms may be mild in the early stage of TB, they are easily ignored by patients. This can contribute to the delay in seeking care. In addition, because of individual factors and others pertaining to the health care system, delays in care seeking, diagnosis and treatment of TB are common.

Recent reports in the literature show that the median delay in care seeking varied from 13 to 30 d and the median delay in diagnosis varied from 15 to 53 d in Australia and other countries.^2,3,5–8^ A study conducted in Iran showed that the median delay in treatment was 35 d.^9^ Delays in care seeking, diagnosis and treatment of TB could increase the risk of adverse outcomes (including exacerbation of the condition, leading to various complications and increasing the risk of death) and result in TB transmission in the community.^10,11^

The delays associated with TB in China are even worse. Data from seven FIDELIS (Fund for Innovative DOTS Expansion through Local Initiatives to Stop TB) projects from 2003 to 2008 in China showed that the median delay in care seeking was 93 d, which is significantly higher than that of other countries.^12^ Furthermore, delays in the diagnosis of TB in different provinces in China vary greatly.^13^ There are significant regional variations in economic development in China and health care resources are unequally distributed across regions.^14^ According to data in the China Statistical Yearbook, Jiangsu province had the highest economic level in China in 2012 (per capita gross domestic product [GDP]=68 347 yuan; number of doctors per 1000 population=1.99), whereas Guizhou province has the lowest (per capita GDP=19 710 yuan; number of doctors per 1000 population=1.41). Hubei province has a moderate economic level (per capita GDP=38 572 yuan; number of doctors per 1000 population=1.89). Studies have shown that economic conditions and accessibility of health care services have a significant impact on TB-related delays.^4,15,16^ However, there has been no relevant research regarding TB-related delays in Hubei. Therefore it is necessary to identify the factors that influence TB-related delays in Hubei.

Identifying the risk factors for delays in care seeking, diagnosis and treatment is the basis for effective control of TB. Several studies have been conducted in China to explore factors associated with delayed TB diagnosis and management. However, most of these studies were conducted among urban migrants, whereas data obtained in rural areas of Hubei province are limited.^17–20^ The socio-economic conditions and accessibility of health care services are relatively different between rural and urban areas. Therefore it is necessary to evaluate rural areas and identify factors associated with delays in the care seeking, diagnosis and treatment of TB.

The purpose of the present study was to evaluate delays among patients with TB in rural areas of Hubei province and to explore factors contributing to delays in care seeking, diagnosis and treatment, thus providing evidence for the prevention and control of TB in rural areas.

## Methods

### Study population

This cross-sectional study was conducted between October 2013 and March 2014 in Hubei province, Central China. Participants were selected using stratified random sampling. First, the 17 cities across Hubei province were divided into three strata (upper, middle and lower) according to their economic development level (per the National Bureau of Statistics), with 1 city randomly selected in each stratum. Second, one district was randomly selected in each selected city. Each district has only one designated institution providing TB diagnosis and treatment services, and all patients with TB who visited the designated institution in the study sites during the study period were recruited.

Inclusion criteria for the participants in this study were as follows: diagnosis of TB according to national TB program guidelines, no psychosis and willingness to participate in the study. For a diagnosis of TB, a case must meet one of the following conditions: two positive sputum smears, one positive sputum smear and one positive sputum mycobacterial culture or one positive sputum smear with typical pathology of active TB on chest radiography.

### Data collection

A structured self-administered anonymous questionnaire was used to collect data on sociodemographic characteristics (sex, age, education, marital status and monthly household income), medical insurance, facility of first consultation (the medical institution where the patient with TB sought care for the first time after onset of any TB-suspicious symptoms such as cough or haemoptysis), facility of diagnosis (the medical institution where the patient was diagnosed with TB), the time required to reach a township hospital by the most commonly used means of transportation and the delay associated with TB.

According to the Tuberculosis Control and Assessment Protocol established by the Chinese Ministry of Health and the existing literature,^21,22^ TB-related delays can be divided into three types: delay in care seeking, delay in diagnosis and delay in treatment, which were measured by three questions (Supplementary Table 1). Delay in care seeking occurred if the time interval between the date of onset of suspicious symptoms of TB and first presentation to a professional health care provider was >3 weeks. Delay in diagnosis occurred if the time interval between the date of first presentation to a professional health care provider and TB diagnosis was >2 weeks. Delay in treatment occurred if the time interval between the date of TB diagnosis and initiation of treatment was >1 week.

### Statistical analysis

All statistical procedures were performed using SAS version 9.3 for Windows (SAS Institute, Cary, NC, USA). First, we carried out descriptive analyses on baseline characteristics of participants and report numerical variables as means and standard deviations (SDs), whereas categorical data are reported as frequencies and percentages. Second, the χ^2^ test was used to explore the association between independent categorical variables and the TB-related delay. Third, the variables with a p-value ≤0.15 in the χ^2^ test were entered into a multivariate logistic regression model to calculate adjusted odds ratios (aORs) for the potential risk factors of delay in care seeking, delay in diagnosis and delay in treatment. All comparisons were two-tailed and p-values <0.05 were considered statistically significant.

## Results

Overall, 1430 participants who completed the questionnaire were enrolled. The total response rate was 93.85%. Of the 1430 questionnaires that were returned, 88 were discarded because of excessive missing data. Thus a total of 1342 patients with TB with a mean age of 47.72 y (SD 17.06) were included in the study. The proportion of patients experiencing delays in care seeking, diagnosis and treatment were 21.54%, 23.62% and 42.25%, respectively. The χ^2^ tests showed that age, education, medical insurance and monthly household income were associated with delays in care seeking; sex, education, medical insurance, monthly household income, time to reach a township hospital and facility of first consultation were associated with delays in diagnosis; and sex, education, marital status, monthly household income, time to reach a township hospital, facility of first consultation and facility of diagnosis were associated with delays in treatment (Table 1).

**Table 1.** Participants’ characteristics and their associations with delay in care seeking, diagnosis and treatment

Variable	Total		Delay in care seeking		p-Value	Delay in diagnosis		p-Value	Delay in treatment		p-Value
	n	%	n	%		n	%		n	%	
Total	1342	100.00	289	21.54		317	23.62		567	42.25	
Age (years) (missing=14)					0.0392*			0.3724			0.5380
11–29	283	21.31	50	17.67		56	19.79		129	45.58	
30–44	231	17.39	42	18.18		53	22.94		99	42.86	
45–59	440	33.13	95	21.59		107	24.32		178	40.45	
≥60	374	28.16	97	25.94		95	25.40		153	40.91	
Sex					0.3138			0.0305*			0.0012*
Male	905	67.44	202	22.32		198	21.88		355	39.23	
Female	437	32.56	87	19.91		119	27.23		212	48.51	
Education					0.0811*			0.0768*			0.0347*
Primary or less	574	42.77	139	24.22		153	26.66		222	38.68	
Secondary	540	40.24	110	20.37		116	21.48		235	43.52	
High school or higher	228	16.99	40	17.54		48	21.05		110	48.25	
Marital status (missing=33)					0.2421			0.9369			0.0894*
Married	1030	78.69	233	22.62		246	23.88		418	40.58	
Other	279	21.31	54	19.35		66	23.66		129	46.24	
Medical insurance (missing=12)					0.0637*			0.0259*			0.2204
Yes	69	5.19	21	30.43		24	34.78		34	49.28	
No	1261	94.81	265	21.02		291	23.08		527	41.79	
Monthly household income (yuan) (missing=236)					<0.0001*			0.0217*			0.0075*
0–999	337	30.47	98	29.08		91	27.00		137	40.65	
1000–1999	309	27.94	54	17.48		91	29.45		145	46.93	
≥2000	460	41.59	78	16.96		97	21.09		164	35.65	
Time to reach township hospital (minutes) (missing=29)					0.3256			<0.0001*			<0.0001*
0–29	661	50.34	132	19.97		105	15.89		242	36.61	
30–59	469	35.72	108	23.03		138	29.42		208	44.35	
≥60	183	13.94	44	24.04		65	35.52		103	56.28	
Facility of first consultation (missing=8)								<0.0001*			0.0070*
TB dispensary or TB unit under the CDC	275	20.61				41	14.91		98	35.64	
Primary medical system	726	54.42				209	28.79		332	45.73	
Hospital	333	24.96				63	18.92		130	39.04	
Facility of diagnosis (missing=8)											<0.0001*
TB dispensary or TB unit under the CDC	790	59.22							284	35.95	
Primary medical system	233	17.47							147	63.09	
Hospital	311	23.31							131	42.12	

*p<0.15.

Multivariate logistic regression analysis indicated that patients with a higher income were less likely to have increased delays in care seeking than patients with a low income. Medicare insurance was a protective factor against delays in care seeking. Moreover, patients who spent more time traveling to a township hospital were more likely to experience delays in diagnosis and treatment. Taking TB dispensaries or TB units under the Center for Disease Control and Prevention (CDC) as references, we found that going to a primary health care institution for a first consultation (aOR 2.60 [95% confidence interval {CI} 1.71 to 3.96]) was a risk factor for delays in diagnosis, and being diagnosed with TB in a primary health care institution (aOR 2.85 [95% CI 2.00 to 4.07]) was a risk factor for delays in treatment. Furthermore, female sex (aOR 1.51 [95% CI 1.15 to 1.99]) was significantly associated with higher odds of delay in treatment. Interestingly, in this study, patients with high school or higher education were more likely to experience delays in treatment than patients with primary school or lower education (Table 2).

**Table 2.** Multivariate logistic regression analysis of factors associated with delays in care seeking, delays in diagnosis and delays in treatment

Variable	Delay in care seeking		Delay in diagnosis		Delay in treatment	
	aOR	95% CI	aOR	95% CI	aOR	95% CI
Age (years) (ref: 11–29)						
30–44	1.02	0.60 to 1.72				
45–59	1.05	0.64 to 1.70				
≥60	1.28	0.75 to 2.21				
Sex (ref: male)						
Female			1.12	0.82 to 1.51	1.51	1.15 to 1.99*
Education (ref: primary or less)						
Secondary	1.10	0.76 to 1.58	0.94	0.68 to 1.30	1.22	0.91 to 1.63
High school or higher	1.06	0.62 to 1.80	1.01	0.65 to 1.57	1.60	1.06 to 2.42*
Marital status (ref: married)						
Other					1.18	0.84 to 1.66
Medical insurance (ref: yes)						
No	1.84	1.01 to 3.38*	1.79	0.97 to 3.28		
Monthly household income (yuan) (ref: 0–999)						
1000–1999	0.53	0.36 to 0.79*	1.19	0.82 to 1.71	1.30	0.93 to 1.83
≥2000	0.52	0.36 to 0.75*	1.01	0.70 to 1.46	0.93	0.67 to 1.29
Time to reach township hospital (minutes) (ref: 0–29)						
30–59			1.78	1.28 to 2.48*	1.37	1.03 to 1.84*
≥60			2.23	1.49 to 3.35*	2.16	1.48 to 3.14*
Facility of first consultation (ref: TB dispensary or TB unit under the CDC)						
Primary medical system			2.60	1.71 to 3.96*	1.07	0.75 to 1.53
Hospital			1.26	0.76 to 2.07	1.14	0.74 to 1.75
Facility of diagnosis (ref: TB dispensary or TB unit under the CDC)						
Primary medical system					2.85	2.00 to 4.07*
Hospital					1.25	0.88 to 1.78

*p<0.05.

## Discussion

This study showed that in rural areas of Hubei province, 21.54%, 23.62% and 42.25% of patients with TB experienced delays in care seeking, diagnosis and treatment, respectively, and these values were lower than those recorded in other provinces in China.^13,23^ In this study, only 22% of patients experienced delays in care seeking, which may be related to the strategy of most national TB programs recommending consultation after a prolonged cough of only 2 weeks. Furthermore, the percentages of patients experiencing delays in the present study were lower than those reported in most countries worldwide. For example, 68.7% patients with TB in Ethiopia experienced delays in diagnosis and 42.3% patients with TB in India experienced treatment delays.^24,25^ Nevertheless, there is still a lot of room for improvement in China.

The proportion of patients experiencing delays in treatment was very high in this study, even though they had been diagnosed with TB. This may be because TB treatment is provided only by designated TB institutions in China and therefore patients with TB diagnosed in other institutions must travel to a designated TB institution for treatment, which makes them more likely to experience delays in treatment.

This study confirmed the impact of the accessibility of health care services on the use of health care services. Patients who spent more time to reach a township hospital were more likely to experience delays in diagnosis and treatment, which was consistent with previous reports.^6,26^ However, it is worth noting that the time to reach a township hospital was not related to delays in care seeking. In fact, in the rural areas, village clinics often serve as the default access institution for rural residents when they have a mild illness. However, there is no equipment for diagnosing TB in village clinics, and patients with TB have to go to township hospitals or designated TB institutions for diagnosis and treatment. Therefore the time to reach a township hospital was not significantly associated with delay in care seeking but was significantly associated with delays in diagnosis and treatment. On the other hand, there was a significant increase in the risk of delays in care seeking among patients with a low economic level, which reflected the effect of affordability on whether the need for health care services can be transformed into the use of health care services. Interestingly, economic factors were not significantly associated with delays in treatment for TB. This might be because of the policy of free treatment for all patients with TB implemented by the Chinese government. Patients can get free anti-TB drugs once they have been diagnosed with TB, which encourages patients to seek timely treatment.

In the present study, delays in diagnosis were more common among patients who chose primary health care institutions for their first medical consultation. This might be because in primary health care institutions, the common symptoms of TB, such as cough and fever, are easily misdiagnosed as other common diseases such as colds, which might result from the lack of necessary medical equipment and specialist doctors in rural areas. Considering that more than half of the patients with TB in this study had their first medical consultation at a primary health care institution, the capabilities of primary health care institutions in rural areas to diagnose and treat TB must be improved. In addition, this study also found that patients diagnosed with TB in institutions not designated to treat TB had a higher risk of delays in treatment, which might be related to China’s TB prevention and control policy. In China, patients with TB can receive free medications only in designated institutions (TB dispensary or TB unit under the CDC) after a diagnosis of TB. Although a tracking and management information system has been implemented in Hubei province to supervise and urge patients with TB diagnosed in undesignated institutions to receive timely treatment in designated institutions, some patients with TB were not tracked in time because of a lag in information transfer from undesignated institutions, thus resulting in delays in treatment. Moreover, the limited knowledge of TB and the lack of understanding of the national free treatment policy for TB were also potential risk factors for delays in treatment. Therefore it is necessary to optimize the tracking management information system to enhance the timely tracking and treatment of patients with TB. At the same time, public health education should be strengthened to enhance public recognition of the importance of national TB prevention and control policies and timely treatment.

The impact of basic demographic characteristics on TB-related delays varied greatly because of differences in social and cultural backgrounds. This study showed that women were more likely to experience delays in treatment. In China, the economic status of women is lower than that of men, which may be one of the reasons women are more likely to experience delays in treatment. This result indicated that in the rural areas of China, the health care needs of women might be neglected and the equity of health care service utilization should be improved. However, age was not significantly associated with TB-related delays, which differed from the results of a study conducted in Myanmar showing that age >30 y was a risk factor for treatment delays.^27^ In the present study, patients with a high educational level had a greater likelihood of delays in treatment, which differed from the reports of other studies and requires further research.^28,29^

This study had some limitations. First, although we took various measures to control the quality of the field investigation, recall bias was hard to avoid as this was a retrospective study. Second, all the participants were patients with TB registered in designated institutions. However, there were still some patients with undiagnosed TB as well as those who had been diagnosed but refused to receive treatment and examination in designated institutions, who were more likely to experience TB-related delays.

## Conclusions

Delay in care seeking, diagnosis and treatment of patients with TB in the rural areas of China remains a serious issue, especially delays in treatment. The Chinese government should strengthen the national policy to provide free TB treatment. Optimizing the process of tracking the management of patients with TB and encouraging patients to accept standardized treatment in a timely manner are imperative. In addition, attention should be paid to the use of health care services among women in rural areas.

## Supplementary Material

ihz036_Supplementary_table
