# Supplementary material for: Delays in care seeking, diagnosis and treatment of patients with pulmonary tuberculosis in Hubei, China
Source: Int Health. 2019 Jun 22;12(2):101–6. doi: 10.1093/inthealth/ihz036 (PMC11700883; doi:10.1093/inthealth/ihz036)
Supplement: ihz036_Supplementary_table [file inthealth_12_2_101_s1.docx]

**Supplementary table 1. TB Related-Delay Questionnaire.**

Instructions: Below is a list of the TB-related delay. Please choose the answer according to your actual situation.

| 1 | what is the time interval between the date of onset of suspicious symptoms of TB (e.g. cough, hemoptysis, night sweat, fever, and chest pain) and your first presentation to a professional health provider? |
| --- | --- |
|  | A. < 1 week; B. < 2 weeks; C. < 3 weeks; D. 3 weeks - 2 months; E. 2-3 months; F. >3 months |
| 2 | What is the time interval between the date of your first presentation to a professional health provider and being diagnosed as TB? |
|  | A. < 1 week; B. < 2 weeks; C. < 3 weeks; D. 3 weeks - 2 months; E. 2-3 months; F. >3 months |
| 3 | What is the time interval between the date of TB diagnosis and initiation of treatment? |
|  | A. < 1 week; B. < 2 weeks; C. < 3 weeks; D. 3 weeks - 2 months; E. 2-3 months; F. >3 months |
